# Thyrotropin-Releasing Hormone and Food Intake in Mammals: An Update

**DOI:** 10.3390/metabo14060302

**Published:** 2024-05-26

**Authors:** Yamili Vargas, Ana Elena Castro Tron, Adair Rodríguez Rodríguez, Rosa María Uribe, Patricia Joseph-Bravo, Jean-Louis Charli

**Affiliations:** Departamento de Genética del Desarrollo y Fisiología Molecular, Instituto de Biotecnología, Universidad Nacional Autónoma de México (UNAM), Avenida Universidad 2001, Cuernavaca 62210, Mexico; yamili.vargas@ibt.unam.mx (Y.V.); elena.castro@ibt.unam.mx (A.E.C.T.); adair.rodriguez@ibt.unam.mx (A.R.R.); rosa.uribe@ibt.unam.mx (R.M.U.); patricia.joseph@ibt.unam.mx (P.J.-B.)

**Keywords:** TRH, hypothalamus, arcuate nucleus, lateral hypothalamus, nucleus accumbens, raphe nuclei, brain stem, TRH-R1, TRH-DE, food intake

## Abstract

Thyrotropin-releasing hormone (TRH; pGlu-His-Pro-NH2) is an intercellular signal produced mainly by neurons. Among the multiple pharmacological effects of TRH, that on food intake is not well understood. We review studies demonstrating that peripheral injection of TRH generally produces a transient anorexic effect, discuss the pathways that might initiate this effect, and explain its short half-life. In addition, central administration of TRH can produce anorexic or orexigenic effects, depending on the site of injection, that are likely due to interaction with TRH receptor 1. Anorexic effects are most notable when TRH is injected into the hypothalamus and the nucleus accumbens, while the orexigenic effect has only been detected by injection into the brain stem. Functional evidence points to TRH neurons that are prime candidate vectors for TRH action on food intake. These include the caudal raphe nuclei projecting to the dorsal motor nucleus of the vagus, and possibly TRH neurons from the tuberal lateral hypothalamus projecting to the tuberomammillary nuclei. For other TRH neurons, the anatomical or physiological context and impact of TRH in each synaptic domain are still poorly understood. The manipulation of TRH expression in well-defined neuron types will facilitate the discovery of its role in food intake control in each anatomical scene.

## 1. Introduction

Thyrotropin-releasing hormone (TRH; pGlu-His-Pro-NH2) is a small peptide produced in neurons and other cells through proteolytic processing of a large precursor [1,2] expressed mainly in the central nervous system (CNS) but also in some peripheral locations. It is an intercellular signal with many actions. Hypophysiotropic TRH neurons in the paraventricular nucleus of the hypothalamus (PVH) integrate information about energy balance to regulate thyrotropin secretion and thus thyroid hormone (TH) secretion [3,4]. Additionally, mRNA coding for pre-pro-TRH, pro-TRH, TRH, and other pro-TRH-derived peptides are detected in various regions of the brain and a few other organs [5,6,7,8,9,10]. Among its multiple pharmacological effects, that on food intake is still puzzling. Since food intake is usually modified in obesity [11], knowledge of the mechanisms involved in TRH could lead to novel treatments for this condition. Although some reviews have included the effect of TRH on food intake [12,13,14,15,16,17,18,19], the understanding of its mechanisms of action is far from consensus. In this manuscript, we review the original literature on the role of TRH in food intake. We focus mainly on functional and anatomical studies, excluding studies that do not precisely indicate the circuit under analysis, and most studies describing correlative evidence. Thus, based on recent advances in the circuits that control food intake, TRH cell and receptors’ cartographies, and functional effects of TRH in multiple anatomical sites, we present a perspective that addresses hypotheses about TRH circuits that are involved in food intake control. The next section refers briefly to some of the core mechanisms to introduce the context of TRH studies on food intake.

## 2. Food Appetite, Foraging, Intake, Satiation, and Satiety

**Figure 1:**
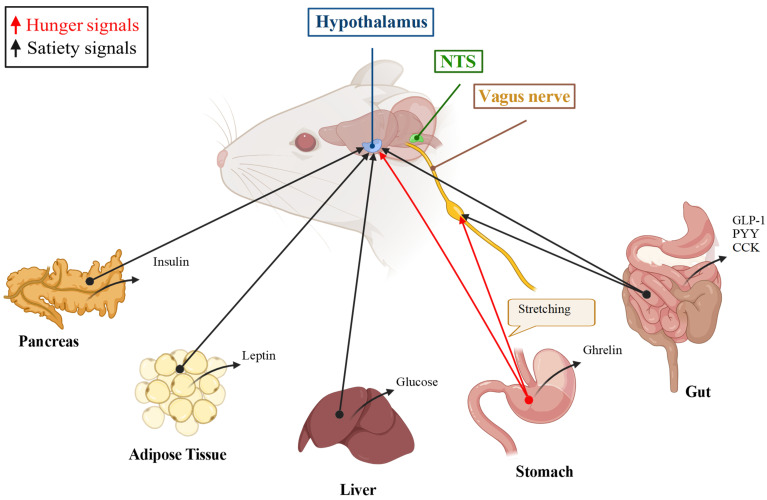
Peripheral signals and central nodes that control food intake. The peripheral organs produce signals related to energy stores and recent meals, signals that promote satiety (black arrows) or hunger (red arrows). These signals can act in circumventricular organs or cross the blood–brain barrier to act in the hypothalamus or nucleus of the solitary tract (NTS) or are perceived by the vagus nerve. Both the hypothalamus and the NTS integrate these signals with central clues to generate the behavioral repertoire of food intake. GLP-1: glucagon peptide-like 1, PYY: peptide YY, CCK: cholecystokinin. Detailed information can be found in references [20,21,22,23,24,25,26,27,28,29,30,31,32]. The figure was created with BioRender.com.

The brain and periphery interact through multiple circuits to control energy intake (Figure 1). Many reviews have dealt with this actively researched field [20,21,22,23,24,25,26,27,28,29,30,31,32]. Energy intake is a complex behavior that goes from motivation for food intake, to food foraging, and consummatory episodes. Foraging and food intake are induced by hunger and by appetite, psychological experiences related to the energy state of the organism, and emotional or external causes. Food intake leads to reward and satiation, and thus intake termination, and a state of satiety. After food consumption, satiation occurs when feeling full or satisfied; it dictates the end and therefore the size of the meal and translates the recent postprandial record. On the other hand, during postprandial fasting, satiety indicates the absence of desire for food and determines the intervals between meals [33].

Food intake depends on circadian, anticipatory, learnt, sensory, and hedonic clues as well as peripheral signals of energy homeostasis interacting with brain circuits encoding the behaviors needed to acquire and consume food, most of it below our conscious awareness. Energy intake is controlled by short-term mechanisms and over extended periods of time through a homeostatic system. Integration of systemic metabolic information with sensory and hedonic information allows the initiation/prolongation or termination of food consumption according to sensory and external information independently of homeostatic signals. Thus, emergency circuits can override homeostatic circuits and promote or inhibit food intake [34].

Food intake behavior involves a neural network organized around key nodes, such as the nucleus of the solitary tract (NST), the hypothalamus, the parabrachial nucleus (PBN), as well as the amygdala, the striatum, and cortical areas [34,35]. Peripheral signals (nutrients, hormones, cytokines) communicate information about acute nutritional state, energy stores, and inflammatory status to the brain. Physical information from the gastrointestinal tract (GI) is furthermore detected by vagal afferent fibers which have a connection with the NTS in the caudal hindbrain [34,36].

In addition to being the target of vagal afferents that transmit peripheral information, neurons of the NTS are next to the area postrema, a circumventricular organ that is outside the blood–brain barrier (BBB) and receives information directly from humoral factors, which is then transmitted to the NTS. This allows the NTS to control short-term homeostasis, through projections to ingestive premotor neurons in the hindbrain and to the hypothalamus, including the arcuate nucleus of the hypothalamus (ARC) [24,37].

The ARC is critical for sensing energy balance [34] because, in addition to receiving input from various brain nuclei, it is adjacent to the median eminence of the hypothalamus (ME), a circumventricular organ, and partially outside the BBB, which facilitates directly receiving energy-related clues. ARC has two main neuronal populations involved in food intake control: one type that synthesizes neuropeptide Y (NPY) and co-expresses Agouti-related protein (AgRP), as well as gamma-aminobutyric acid (GABA), stimulates the appetitive and consummatory aspects of food intake behavior; and a second type that synthesizes pro-opiomelanocortin (POMC), the precursor of α- or β-melanocortin (α- or β-MSH), and co-expresses cocaine and amphetamine-regulated transcript (CART), has a potent appetite-suppressing activity preferentially relevant for long-term control of food intake [37,38,39,40,41].

NPY/AgRP/GABA^ARC^ and POMC/CART^ARC^ neurons project to hypothalamic and extrahypothalamic neurons that control reward and food intake. Among these targets, PVH is critical for food intake; for example, activation of AgRP terminals in PVH reproduces the food intake observed when stimulating AgRP neurons in the ARC [41]. Another target of ARC is the dorsomedial nucleus of the hypothalamus (DMH), a positive regulator of the circadian control of food intake behavior [42].

A critical downstream target of the ARC is the lateral hypothalamus (LH), which integrates reward and energy homeostatic information and generates outputs to midbrain motor pattern generators that maintain the behavioral repertoire of food intake, i.e., the mesolimbic dopaminergic system, as well as the NTS, which regulates satiety [20,21,43,44,45,46,47,48]. The LH contains orexin (ORX) neurons, critical for the promotion of food intake [49,50], as well as melanin-concentrating hormone (MCH) neurons that promote appetite and consumption [51], and many GABA neurons that form functionally diverse subpopulations regulating food intake [52,53].

Furthermore, projections from NPY/AgRP/GABA^ARC^ neurons to the PBN are critical for food intake control [54,55]. The PBN also receives direct input from two glutamatergic^PVH^ neuron types that control food intake [56,57].

The ARC neurons also project to the reward circuits including mesolimbic dopaminergic neurons of the ventral tegmental area (VTA) that project to the nucleus accumbens (NAc) and other regions. For example, a circuit between AgRP neurons and mesolimbic dopamine neurons regulates food reward [58]. In the nucleus accumbens shell (NAcSh), dopamine communication integrates motivational and sensory inputs, is involved in incentive salience [59,60], and when activated reduces food intake [61,62,63,64,65,66]. Dopamine receptor 1^NAcSh^ neurons project onto GABA^LH^ neurons that tend to be in apposition with ORX or MCH neurons and transmit a stop-eating signal when they are activated [67].

Research on food intake has allowed the visualization of some of the major circuits and molecular mechanisms that control multiple outputs; however, several questions remain unanswered. Among them, the effect of TRH on food intake, albeit identified a few decades ago, is still poorly understood.

## 3. Discovery of the Effects of TRH, a TRH Analogue, and TRH Catabolites on Food Intake

### 3.1. The Peripheral Administration of TRH Modifies Food Intake in Mammals, According to the Route of Administration

Food intake increases in female rats consuming TRH in drinking water over 30 days (Supplementary Material Table S1). TRH may be transported through the intestinal epithelial barrier by peptide transporter 1 (SLC15A1), one of the proton-coupled oligopeptide transporters, which is expressed in the intestine and can transport TRH [68]. TRH may then enter the general circulation and act through the pituitary–thyroid axis since the effect on food intake is blocked by thyroidectomy (Table S1). This route of administration may lead to the preferential activation of anterior pituitary TSH secretion and TH-regulated food intake.

In rodents, the peripheral or intracerebral injection of 3,3′,5-triiodothyronine (T3) increases food intake, possibly through regulation of either NPY^ARC^ and POMC^ARC^ neurons, although this has been contested, and/or through regulation of the phosphorylation of 5’ adenosine monophosphate-activated protein kinase in the ARC, or through a direct effect of T3 on the ventromedial hypothalamus (VMH) (Table S2).

In contrast to its effect in drinking water, other reports indicate a strong anorexic effect of TRH if administered through subcutaneous (sc) or intraperitoneal (ip) routes. After one TRH injection, the effect is maximal after 15–30 min, disappearing after 1 h. If a chronic administration is used, the results are less consistent. The data are similar in rats, mice, Siberian hamsters, and dogs. They are observed in either genetically obese or wild type rats fed ad libitum, or in tail-pinched or starving rats (Table S1) [69]. Although the phase of food intake behavior affected by TRH cannot be ascribed, some of the data suggest that there is, at least in part, an effect on the consummatory phase. However, the precise impact of TRH on this phase of food intake (food intake delay, meal frequency, duration, etc.) is clearly missing.

### 3.2. TRH Acts through TRH Receptor-1 (TRH-R1) and TRH Receptor-2 (TRH-R2) in Mammals

Three subtypes of TRH receptors (TRH-R1-3), that are closely related to GTP-binding protein-coupled receptors (GPCRs), have been characterized. Mammals generally express *Trhr* (TRH-R1) and *Trhr2* (TRH-R2); humans express *Trhr* and a receptor distinct from *Trhr* whose sequence awaits characterization [70].

When activated by TRH, TRH-R1 and TRH-R2 exhibit similar signaling pathways. In mammals, TRH effects are transduced through Gq/11, as well as Gs and Gi subunits, with the intracellular pathways and the signaling outcome depending on the cell type where TRH-R is present; TRH-R activation leads to desensitization and internalization of the receptor [71,72]. For example, the recurrent application of TRH to neurons of the dorsal motor nucleus of the vagus (DMV) promotes a reduction in the response [73].

TRH receptors are expressed mostly in endocrine cells and neurons. In neurons, TRH receptor activation is linked to the modification of cation conductance mediated by either G protein-coupled inwardly rectifying potassium channel-like proteins, or transient receptor potential channel-4/5, or fast transient A-type potassium current, or calcium-dependent slow after-hyperpolarization, or other channels, and generally stimulates neuronal excitability through a postsynaptic action [74,75,76,77,78,79,80].

In the central nervous system, *Trhr* expression is high in the hypothalamus and brainstem, while that of *Trhr2* is more extensive, including the thalamus, the cerebral and cerebellar cortex, medial habenula, medial geniculate nucleus, pontine nuclei, and reticular formation [81]. In peripheral tissues, *Trhr* mRNA is found in the heart, spleen, liver, lung, skeleton, muscle, kidney, testis, stomach, small intestine, colon, adrenal medulla, and pancreas [71,82,83]. In contrast, *Trhr2* has a limited peripheral distribution; it is present in the testis and gastrointestinal tract [71,82].

There is a lack of specific antagonists of TRH receptors. In vitro, a benzodiazepine working at micromolar concentration has been used to test whether TRH effects can be attributed to interaction with its receptors [84], but the specificity and potency of this benzodiazepine are not adequate for in vivo studies. Recent results indicate that [β-Glu2]TRH is a functional antagonist of TRH-R1 [85], yet to be tested in food intake experiments.

### 3.3. Peripheral TRH Is Hydrolyzed by a Metallopeptidase That Likely Limits Its Effect on Food Intake

The peripheral effect of TRH is transient, likely because of receptor desensitization, but also due to peptide inactivation through renal clearance [86] or hydrolysis. TRH degradation half-life in plasma or in blood after intravenous (iv) injection is of a few minutes [87,88]. The major mechanism of extracellular inactivation of TRH is its hydrolysis by thyrotropin-releasing hormone-degrading ectoenzyme (TRH-DE), a narrow specificity enzyme whose main biological substrate is TRH. Apart from the brain, the enzyme is detected in serum and at low levels in some peripheral organs [17]. Hydrolysis of TRH by TRH-DE produces histidyl-proline amide, which spontaneously cyclizes to his-pro-diketopiperazine (HPD), and pyroglutamic acid. The intraperitoneal injection of pyroglutamic acid or HPD to adult male rats does not change food intake (Table S1).

Since the anorectic effect of peripheral TRH on food intake takes minutes to develop, the mechanisms involved should be faster than those relying on long-term effects of TRH, such as cell survival. In the remainder of the review, we will focus only on mechanisms that are sufficiently rapid to contribute to the TRH effect.

### 3.4. The Anorexic Effect of Peripheral TRH on Food Intake Is Independent of the Control of the Pituitary–Thyroid Axis

Transient activation of the pituitary by peripheral TRH may lead to effects on food intake. The peripheral effect of TRH on food intake occurs before it produces any effect on serum TH concentrations [89], although changes in serum TSH concentration occur more rapidly. The systemic injection of TRH enhances plasma TSH concentration within 15 min, while levels of TH are not changed before 30 min [90,91,92,93].

A high dose of TSH administered intraperitoneally (ip) does not inhibit short-term food intake in adult male rats that have been food deprived, but the intracerebroventricular (icv) injection of TSH reduces food intake in rats. Because in mice TSH-R is expressed by α2-tanycytes that send cytoplasmic extensions into the ARC [94], the effect of icv injection of TSH may reflect a central action of TSH produced locally, not related to TRH. Finally, sc TRH-induced suppression of mild tail-pinch-induced eating is detected in hypophysectomized animals, showing that it is not a TSH- or TH-dependent effect (Table S1). This evidence is consistent with the idea that the anorexic effect of peripheral TRH cannot be mediated through an increase in TSH or TH.

### 3.5. Peripheral TRH Effect on Food Intake and Vagus Nerve or Other Sensory Nerve Inputs

The peripheral effect of TRH on food intake requires an intact vagus nerve [95], suggesting the engagement of a peripheral TRH receptor whose activation generates an anorectic signal conveyed through the vagus nerve to the brain stem. An alternative interpretation is that the activity of the vagus nerve is required to express the effect of TRH on food intake [96].

Multiple single cell transcriptome studies reveal a high heterogeneity of cell molecular signatures in the peripheral nervous system (PNS) [97]. Three major PNS neuronal types, sensory (dorsal root ganglia), sympathetic, and enteric neurons, subclassified in clusters according to the expression of multiple molecular markers [98] did not show expression of *Trhr*. However, the integration of recent and focused organ transcriptomes shows *Trhr* expression in a cluster corresponding to *Gabra* expressing neurons from the nodose ganglia [99], coincident with data showing that TRH enhances intracellular Ca^2+^ concentration in a subset of neurons of the nodose ganglion in cell culture [100]. Whether these neurons convey an anorexic signal to the brain awaits resolution, as with the endogenous source of TRH acting on the nodose ganglion. Since circulating levels of TRH are very low [101], it is unlikely that an effect on the ganglion is due to an endocrine source of TRH.

### 3.6. Peripherally Injected TRH Can Enter the Brain through the BBB

Although initially a controversial concept, it is now clear that peptides can cross the BBB through various mechanisms [102]. Critical characteristics include the molecular weight (lower than 500 Daltons facilitate entry) [103], lipophilicity (the higher the better entry), which allow non-saturable entry, and absolute charge, that can allow adsorptive transcytosis. In addition, some peptides are transported by a saturable system, which seems to be, quantitatively, the main mechanism [102].

Although TRH permeability through the BBB is very low [104], a small fraction of peripherally injected TRH does enter the brain [88]. TRH can be traced in human samples of CSF in biologically active levels after an iv infusion of 10 mg/kg of TRH, peaking after 90 to 120 min post administration [105]. The specific mechanism of TRH entry to the brain is unknown to date, although different measurements show the dynamics of its internalization to the brain. The short-term leakage dynamics of exogenous TRH through the BBB are like other hypophysiotropic peptides and inert polar molecules [106,107]. Experiments in vitro with the blood–brain barrier preparations of sheep, guinea pigs, and rats demonstrate that TRH entry into the brain is a non-selective process in areas like the hippocampus and the cortex, showing no self-inhibitory effects at high concentrations of TRH [107,108]. Observations of the long-term dynamics of TRH unmask a greater uptake of TRH when compared to mannitol [108], which suggests a slow passage of TRH by diffusion in BBB-free areas with the added effect of cerebrospinal fluid bulk flow [106].

Although hypothalamic TRH passage has not been directly measured after peripheral infusion of TRH, a transient passage of TRH into the ventral arcuate nucleus, where adjustments in the plasticity of the BBB structures have been detected during fasting [109,110], and during the circadian cycle [111], should be considered.

The presence of caveolin in the terminal pole of β1- and β2-tanycytes [112] suggests the endocytosis/transport of molecules derived from the blood by a non-clathrin mechanism [113], as with that of other receptor-mediated processes. Thus, an intriguing possibility is brain entry of TRH through transport mediated by TRH receptors in β1- or β2-tanycytes since tanycytes might express *Trhr* [114], but see [115], a mechanism analogous to that suggested for leptin [116] and ghrelin [117] transport in tanycytes. Peripherally injected TRH effects may therefore be due, in part, to a central site of action.

### 3.7. TRH Inactivation In, and Transport Out Of, the CNS Parenchyma

TRH can be hydrolyzed in the brain extracellular space by TRH-DE; thus, the use of TRH analogs resistant to hydrolysis is important [17]. Only one of the hydrolysis-resistant TRH analogs, l-pyroglutamyl-l-histidyl-l-3,3′-dimethyl-prolineamide (RX77368) [118], has been tested in the context of food intake.

TRH could also be removed from the CNS extracellular space by a saturable transport system [119], or by diffusion into the ventricles and subsequent removal by the lymphatic system, and/or by unidirectional brain-to-blood transport [120]. Icv injection of TRH leads to peripheral leakage [121], but it is unlikely that it is relevant to interpret the effect of this on food intake.

### 3.8. The Injection of TRH or of RX77368 in Rat, Hamster, or Mouse Cerebral Ventricles Increases or Reduces Food Intake According to Ventricular Localization

Independently of the peripheral site of action, the intra-lateral ventricle injection of TRH or RX77368 reduces food intake maximally at 0.5–2 h post injection in various models (fasting, tail-pinch- or diazepam-induced eating), species (rat, hamster, or mice), or sex. The effect is dose-dependent and transient. In contrast, icv TRH does not change muscimol- or norepinephrine-induced food intake, suggesting its effect does not occur downstream of GABAA-receptor agonist or norepinephrine effects on food intake. Finally, the food intake reduction induced by ip coadministration of cholecystokinin (CCK) and leptin is reversed by the icv injection of antibodies against TRH and CART, suggesting TRH effect is downstream of leptin and CCK action (Table S3). This is one of the few pieces of evidence that endogenous TRH is relevant in food intake control (see also Section 4.1.5, Section 4.2.2 and Section 4.2.3).

Other data suggest that the sensitivity to the anorexic effect of TRH is localized around the third ventricle (3V) since an intra-3V injection of a very small dose of TRH to rats or Siberian hamsters reduces food intake. In contrast, the intracisternal injection (ic) of RX77368 increases food intake in rats fed ad libitum (Table S3).

Therefore, TRH might control food intake through central sites of action, but since opposing effects are obtained according to the site of action, global approaches might yield confusing results. Mice lacking the *Trh* gene show a transient growth retardation, likely related to the hypothyroid status of these animals [122], but quantification of food intake was not reported. Mice constitutively overexpressing *Trh* in most neurons and other cells have an increased food intake and lower body weight, which is possibly a consequence of increased sympathetic tone and metabolic rate, although thyroid axis hormones have normal serum concentrations [123].

### 3.9. Is the Central TRH Effect on Food Intake Dependent on TRH Receptors, or on a TRH Catabolite?

Because of the lack of specific TRH receptor antagonists, the relevance of TRH receptors for a TRH effect on food intake could be inferred from the use of neutralizing receptor antibodies, antisense tools, the phenotype of mouse knockout (KO) for each receptor, or genome-wide association studies (GWAS). Mice KO for *Trhr* have low serum TH concentrations and mild hyperglycemia [124,125], but daily food intake in 3–4-month -old animals is not altered if normalized to body weight and even if the animals are made euthyroid [126,127]. While in euthyroid *ob*/*ob* mice leptin injection twice daily for three consecutive days powerfully reduces food intake, it does it less conspicuously in hypothyroid *Trhr*/*ob* double KO as well as in 2-mercapto-1-methylimidazole-, sodium perchlorate-treated *ob*/*ob* mice, suggesting that food intake in response to leptin depends on thyroid status but not on TRH-R1 signaling [126]. In fasted *Trhr* KO mice, increases in stomach ghrelin-O-acyltransferase (GOAT) expression and acyl-ghrelin serum concentration are blunted independent of the thyroid state, suggesting TRH-R1 regulates GOAT expression, and thus the concentration of acylated ghrelin in the circulation [127]. Since acylated ghrelin exerts a strong orexigenic effect by activating NPY/AgRP^ARC^ neurons [128,129], these data suggest that TRH-R1 is implicated in a circuit relevant for food intake (see Section 4.2.3). GWAS indicates that *Trhr* is important for lean body mass [130,131], power in athletes [132], and possibly BMI [133,134], but there are no links of *Trhr* to food intake.

The physiological significance of TRH-R2 for central TRH action has been challenged since data in mice show that TRH-R1 is the only receptor relevant for various pharmacological actions of TRH, although food intake was not tested [135]. Mice KO for TRH-R2 have normal body weight, serum TH concentration, and latency and duration of food intake [136], suggesting that TRH-R2 is not critical for food intake.

A less likely alternative is that the anorexic effect of TRH is mediated by a product of the extracellular catabolism of TRH by TRH-DE. HPD has been detected in the CNS [137,138], and at least in some brain regions, a significant percentage seems to arise from TRH catabolism [139]. Icv HPD decreases ad libitum food intake, food deprivation- and stress-induced food intake in rats for various hours, but these results have been attributed to a contamination of the HPD batch (Table S3). Furthermore, many effects of TRH are amplified if analogs resistant to degradation are used, or if inhibitors of TRH-DE are injected [17,140], and because the anorectic effect of TRH is transient while that of HPD is much more persistent (Table S3), it is unlikely that catabolism of TRH is required to obtain an effect on food intake. Finally, young adult mice KO for *Trhde* grown in standard conditions have normal body weight, but food intake was not reported [141].

## 4. In Search of the Central Circuits Involved in TRH Action on Food Intake Behavior

TRH neurons and TRH receptors are localized in various regions related to food intake, and, in some cases, the chemical phenotypes of cells expressing TRH receptors have been identified as well as the electrophysiological, autonomic, endocrine, and behavioral effects of TRH application. It should be noted that, in general, the diffusion range of TRH injected into central nuclei is unknown, making the spatial interpretation of the in vivo pharmacological results imprecise. The evidence suggests that TRH effects on food intake are at least in part due to central interactions. Except for one or two cases, the TRH neuron types that could sustain the physiological equivalent of the pharmacological effects have not been identified. In this section, we evaluate the most promising alternatives.

### 4.1. Putative Hypothalamic TRH Neurons and Targets Sustaining Effect of TRH on Food Intake

#### 4.1.1. Sim1^PVH^ Neurons

Are TRH^PVH^ neurons a relay between ARC neurons and brain neurons that control food intake? The single-minded1 (*Sim1*) gene encodes a transcription factor necessary for the development of the neurons of the PVH [142]. *Sim1*^PVH^ neurons inhibit food intake, at least on a long-term basis [143,144]. Ablation of *Sim1*-expressing neurons or reduction in *Sim1* expression causes similar decreases in *Sim1* and *Trh* expression in the PVH [145,146], suggesting that some *Sim1* neurons are *Trh* neurons, and that most *Trh* neurons are *Sim1* neurons. However, whether *Trh/Sim1*^PVH^ neurons are relevant for the control of food intake is not settled. TRH^PVH^ neurons can be divided into at least two broad types (neuroendocrine or hypophysiotropic, and non-neuroendocrine) [147], and probably into more subtypes [148].

#### 4.1.2. Hypophysiotropic TRH^PVH^ Neurons

The hypophysiotropic neurons of the PVH project their axons into the median eminence and release their neurotransmitters near the portal vessels that irrigate the anterior pituitary. In the rat, the hypophysiotropic TRH neurons are concentrated in the mid-caudal PVH; they express leptin receptors and receive afferents from various limbic regions, adrenergic/noradrenergic fibers from the brain stem, and NPY/AgRP/GABA^ARC^ and POMC/CART^ARC^ neurons, making them able to regulate thyroid economy in response to changing energy levels [3,4,149]. Based on correlative arguments, some authors [18,19] propose that TRH^hypophysiotropic PVH^ neurons are the vector of the anorectic effect of TRH, but there is no concrete (functional) evidence that this is indeed the case.

#### 4.1.3. TRH^anterior PVH^ Neurons

In rodents, most non-hypophysiotropic TRH neurons are concentrated in the anterior PVH (aPVH) [150,151,152]. In rats, TRH^aPVH^ neurons are innervated by NPY/AgRP/GABA^ARC^ and POMC/CART^ARC^ neurons [3] and by adrenergic/noradrenergic fibers from the brain stem [153]. *Trh*^aPVH^ neurons have been associated with anorexia since in adult female Wistar rats, *Trh* expression increases in this part of the PVH in dehydration-induced anorexia [154]. Interestingly, projections of TRH^aPVH^ neurons have been mapped to nuclei relevant for food intake control [155].

The ARC has a TRH innervation [156] arising, at least in part, from the aPVH [155] and expresses both *Trhr* and *Trhr2* [81,157,158]. In mice, an orexigenic (through glutamate) glutamatergic^PVH->ARC^ projection expresses *Trh* [159], but the precise location of the PVH neurons projecting onto the AgRP neurons and the specific role of TRH in this projection remain unknown. This projection regulates the strength of transmission across glutamatergic TRH^PVH^/AgRP synapses, and its glutamatergic activity produces a long-term increase in food intake [160]. In slices, TRH does not affect the membrane potential or spontaneous spiking of POMC and NPY neurons [161]; however, see [162].

TRH terminals are detected in the DMH [163], where a significant population of cells expresses *Trhr* [158]. In rats accustomed to a daily 4 h food intake and drinking schedule, the bilateral injection of 8 nmoles of TRH per hemisphere in the medio-basal hypothalamus (centered around the DMH, although the precision of the procedure is insufficient to be categorical about DMH relevance) produces a sustained (maximum at 30 min, still significant at 3 h reduction in food intake in 20 h food- and water-deprived male adult rats [164]. Prodynorphin^DMH^ neurons express *Trhr*, project into the PVH, and when activated, inhibit food intake [165].

#### 4.1.4. TRH^rostral perifornical LH^ Neurons

The LH contains a large population of TRH neurons that are heavily contacted by axons from the AgRP^ARC^ and POMC^ARC^ neurons [163]. These neurons are localized in the perifornical, tuberal, and peduncular regions of the LH. In rat brain, almost all urocortin 3 (Ucn3) neurons in the rostral perifornical area express *Trh* [166]. ARC dorsomedial and lateral parts receive, respectively, a dense and moderate TRH innervation from, in part, the perifornical area [155]; preferably in the lateral part, many of these TRH fibers are UCN3/TRH axons [166], and more than half of the POMC^ARC^ neurons are in contact with UCN3/TRH axons, which form excitatory synapses. TRH prevents the depolarization and increased firing rate of POMC neurons induced by UNC3 [162].

TRH^perifornical^ neurons also innervate the DMH [155]. The highest density of UCN3/TRH fibers is found in the rostral part of the DMH, primarily in its ventral part [166]. See Section 4.1.3 for a possible consequence of this innervation.

TRH^perifornical^ neurons also project to the VMH [155], which contains a very high density of UCN3/TRH axons, especially in its dorsomedial part [166]. TRH decreases food intake when injected into the medio-basal hypothalamus [164], a region including the VMH, but the precision of the injection is insufficient to be categorical about the target region, and the density of TRH receptors is very low in the VMH [81,158].

#### 4.1.5. TRH^tuberal LH^ Neurons Projecting to Histaminergic Neurons of the Tuberomammillary Nucleus (TMN)

The TMN contains TRH axons that originate, at least in part, in the tuberal LH (TuLH). The TRH^TuLH^ neuron terminals impinge on histaminergic neurons in all subdivisions of the TMN, where approximately half of the histaminergic neurons co-express *Trhr* [167,168]. In histamine-depleted rats, the anorectic effect of icv TRH is reduced [168]. Furthermore, icv anti-TRH antibody suppresses the anorectic action of nesfatin 1, an effect which is histamine mediated [169]. Thus, the control of histamine neurons by TRH^TuLH^ neurons may contribute to the anorectic actions of TRH since histamine neurons control food intake [170].

#### 4.1.6. TRH^DMH^ Neurons Projecting onto LH GABA Neurons That Control MCH Neurons

The DMH is, apart from the PVH, a major site of localization of TRH neurons. Some of the DMH neurons that project to the LH express *Trh* mRNA [42]. TRH terminals [161,163] and *Trhr* expression [81,158] are abundant in the LH. In rats accustomed to a daily 4 h food intake and drinking schedule, the intracranial bilateral injection of 8 nmoles of TRH per hemisphere in the LH does not change food intake in 20 h food- and water-deprived male adult rats [164]. In contrast, other authors show that TRH injection into the LH induces anorexia in rats [171]. The controversy about LH sensitivity to the anorexic effect of TRH has not been settled but may be related to the large extension of the LH.

In LH slices, TRH promotes a reduction in the firing of the MCH neurons; this effect is mainly indirect, through stimulation of the activity of local GABAergic interneurons contacted by TRH neurons, presumably projecting from the DMH. This may contribute to the anorexic effect of TRH [161]. A few data are consistent with the idea that TRH^DMH^ neurons transmit information that is relevant for processing energy balance. Compared to sedentary animals, *Trh* expression in the DMH of male adult rats is enhanced by 2 weeks of voluntary exercise, a condition in which rats consume less than sedentary control animals [172]. Furthermore, in female and male Wistar rats, 2 days of fasting reduce *Trh*^DMH^ expression. In male rats, fasting increases the expression of *Trhr*^LH^ [158], which suggests reduced TRH communication in LH during fasting.

#### 4.1.7. TRH^ARC^ Neurons Projecting Locally 

Scattered cell somata displaying TRH immunoreactivity are observed from bregma −2.3 mm to −3.24 mm in the rat ARC [163]. In mice, afferents to AgRP^ARC^ neurons include GABA/*Trh*^ARC^ neurons, which express the glucagon-like peptide 1 receptor (Glp1r) and are activated by the GLP-1R agonist liraglutide. Activation of GABA/*Trh*^ARC^ neurons inhibits AgRP^ARC^ neurons’ activity and decreases food intake, while inhibition of GABA/*Trh*^ARC^ neurons’ activity increases food intake. The synaptic effects are explained by GABA action on AgRP^ARC^ neurons [173].

### 4.2. Putative Extrahypothalamic TRH Neurons and Targets Sustaining Effect of TRH on Food Intake

TRH neurons acting on food intake are thus clearly localized within the hypothalamus, but this does not exclude that extrahypothalamic TRH neurons are also involved.

#### 4.2.1. TRH^perifornical LH^ and/or TRH^bed nuclei of the stria terminalis^ Neurons That Project to the NAc

The NAc is densely innervated by TRH fibers and terminals [174]. These include a low density of double-labeled TRH/UNC3 fibers likely arising from the perifornical LH and/or bed nucleus of the stria terminalis [166], but the complete map of the TRH neurons innervating the NAc is unknown. In mammals, an intermediate concentration of high-affinity TRH-binding sites is detected in the NAcsh [175], corresponding only to *Trhr* mRNA [81,176].

In rats accustomed to a daily 4 h food intake and drinking schedule, the bilateral injection of TRH in the NAc produces a sustained reduction in food intake. In adult male food-restricted rats, TRH unilateral injection into the NAcsh reduces food intake and motivation to eat, and increases dopamine release from the NAcsh. In ad libitum-fed animals, there is no effect of TRH injection into the NAcsh on food intake. Finally, an intra-NAcsh injection of TRH diminishes chow or palatable food intake in isolation-stressed rats (Table S3).

#### 4.2.2. TRH^NAcsh^ Neurons with Unknown Projections

The NAcsh contains a small density of TRH neurons [81]. Injection of an antisense oligodeoxynucleotide (aODN) against pro-TRH mRNA into the NAcsh of 48 h fasted rats does not change 2 h cumulative food intake but blocks the anorectic effect of α-MSH in the NAc, suggesting that accumbal TRH neurons are downstream of α-MSH actions to inhibit food intake in the NAc. These TRH neuron projections are unknown; since most accumbal neurons are GABAergic medium spiny neurons, they might release TRH in the LH, possibly regulating MCH neurons [177].

#### 4.2.3. TRH^caudal raphe nuclei^ Neurons Innervating the DMV

The physiological significance of this projection has been reviewed [15]. Briefly, TRH neurons located in the raphe pallidus, raphe obscurus, and parapyramidal regions [127,178] innervate neurons of the DMV [179], synapsing on DMV neurons that contribute vagal efferent innervation of the stomach [180,181]. In the DMV, *Trhr* is expressed abundantly in the medial column, which contains neurons that innervate the stomach [81,182,183].

TRH induces a rapid and persistent excitation of these neurons [73,74,184,185], which leads to the enhancement of vagal efferent discharge [186,187,188]. TRH injected ic increases gastric acid secretion through vagal and cholinergic mechanisms [189]. RX77368 ic induces robust cFos expression in the myenteric plexus of the gastric corpus and antrum in conscious fasted rats [190]. In pentobarbital anesthetized rats, ic RX77368 induces total ghrelin secretion through a vagal and atropine-dependent pathway in the stomach. The ic injection of RX77368 stimulates food intake in freely fed rats, an effect that lasts for 3 h, and is inhibited by either peripheral atropine or a ghrelin receptor antagonist. In fasted rats, *Trh*^raphe nucleus^ mRNA expression increases, and food intake is reduced by ic TRH antibody. Thus, the TRH^caudal raphe nuclei^ projection onto the DMV seems to have a physiological role in food intake [191]. Since fasted ghrelin and acyl-ghrelin increases are blunted in *Trhr* KO mice, DMV TRH-R1 might be necessary for the control of ghrelin secretion and food intake [126].

## 5. Conclusions

**Figure 2:**
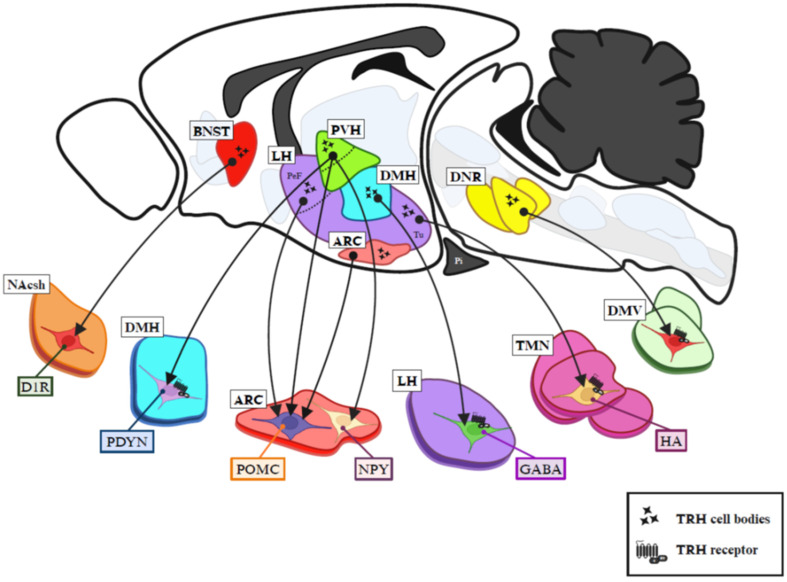
Schematic localization of TRH neurons and projections to intra- and extra-hypothalamic targets putatively involved in food intake control. TRH neurons are represented by black stars; dark arrows indicate their projections. Eight types of TRH neurons are shown. (1) TRH^anterior part of PVH^ (green nuclei, differentiated from the mid/caudal part of PVH by a dotted line) projecting to anorexigenic POMC^ARC^ and orexigenic NPY^ARC^ neurons. (2) TRH^anterior part of PVH^ projecting to PDYN^DMH^ neurons. (3) TRH^ARC^ neurons innervating orexigenic AgRP^ARC^ neurons. (4) TRH^PeFLH^ neurons (purple nuclei, differentiated from LH tuberal area by a dotted line) projecting to anorexigenic POMC^ARC^ neurons. (5) TRH^TuLH^ neurons projecting to histaminergic^TMN^ neurons. (6) TRH^DMH^ neurons projecting to GABA^LH^ neurons. (7) TRH/UNC3^BNST^ neurons projecting to D1R^NAc^ neurons. (8) TRH^caudal raphe nuclei^ neurons innervating DMV neurons that control ghrelin secretion from the stomach. TRH receptor representation is inserted in cells that show strong evidence of TRH receptor involvement in target activation. Abbreviations: ARC, arcuate hypothalamic nucleus; BNST, bed nucleus of the stria terminalis; D1R, dopamine receptor type 1; DNR, dorsal nucleus of raphe; DMH, dorsomedial hypothalamic nucleus; DMV, dorsal motor nucleus of the vagus; GABA, γ-aminobutyric acid; HA, histamine; LH, lateral hypothalamus; NAcsh, nucleus accumbens shell; NPY, neuropeptide Y; PDYN, pro-dynorphin; PeF, perifornical area; Pi, pituitary gland; POMC, pro-opiomelanocortin; PVH, paraventricular hypothalamic nucleus; TMN, tuberomammillary nucleus; Tu, tuberal area. Figure based on [198] and created in BioRender.

The evidence suggests that apart from the putative effect of TRH on the nodose ganglion, TRH effects on food intake are due to interactions with central target cells that express *Trhr* (Figure 2), interactions that are limited by the activity of TRH-DE. However, the pharmacological results should be taken with caution. Non-physiological mechanisms may arise from the fact that the intracerebral injection of TRH or analogues may overstimulate TRH receptors simultaneously in multiple regions and change the balance of action of co-transmitters, generating a non-specific response. In addition, a food intake response could be due to the interference of other behaviors induced by TRH with food intake. Although TRH does not modify some behaviors, such as shuttle box avoidance responding [192], ruling out a generalized non-specific response, it remains possible that arousal or locomotion induction by TRH may interfere with food intake; for example, icv administration of TRH causes behavioral excitation in the rat during a 2 h ingestive period [193]. This idea has been analyzed for the peripheral administration of TRH in the Siberian hamster; the increase in general activity does not affect the time spent eating or near food, suggesting that locomotor activity in response to TRH does not reduce food intake [89], but it will be important to analyze this kind of artefact in each case.

Available data hint at multiple central TRH neuron types and projections as putative controllers of food intake. Confirmatory functional evidence has been obtained in a small number of locations with the use of neutralizing antibodies, chemical depletion of a neurotransmitter, or KO mice. While the orexigenic effect of the TRH^raphe nuclei^ to DMV projection, and possibly the anorexigenic effect of the TRH^TuLH^ to TMN projection, are sustained by functional evidence, in all other cases, the physiological role of TRH in each specific projection is almost unknown.

The effects of TRH on food intake may be carried out by various independent circuits participating in multiple contexts. The physiological events that mobilize each of these circuits are essentially unknown. Some of the TRH projections reviewed above may be the physiological substrate(s) of an anorexic effect of TRH; in one other projection, its role is orexigenic; finally, in yet another set of projections, it is difficult to predict how it will contribute to the control of food intake. It is likely that the complete set of neurotransmitters available in each type of TRH neurons, and the electrophysiological properties of the TRH and target neurons define in each case the physiological relevance of TRH for food intake control.

Other unknowns abound. Most studies have used male adult animals, and thus a critical aspect is to understand the sexual and developmental dependencies of TRH effects. Finally, the evolutionary origin of the effect of TRH is poorly understood. It appears that TRH or TRH-type peptides have the capacity to modulate food intake in many vertebrates and in non-vertebrate deuterostomes [194,195,196,197]. Thus, the control of food intake by TRH-type peptides might have appeared early, even before vertebrate evolution.

## Data Availability

No new data were created or analyzed in this study. Data sharing is not applicable to this article.

## Supplementary Materials

The following supporting information [13,69,95,98,164,168,177,191,192,193,199,200,201,202,203,204,205,206,207,208,209,210,211,212,213,214,215,216] can be downloaded at: https://www.mdpi.com/article/10.3390/metabo14060302/s1, Table S1: Overview of studies regarding the effect of peripheral administration of TRH, TRH analog, and TRH catabolite on food intake behavior in mammals; Table S2: Overview of studies regarding the effect of administration of TSH or thyroid hormones on food intake behavior in mammals; Table S3: Overview of studies regarding the effect of central administration of TRH, its immediate precursor, a catabolite, an analog, an antisense oligonucleotide, or anti-TRH on food intake behavior in mammals.
